# Estrous Cycle Influences Cell-Type-Specific Translatomic Signatures of Repeated Ketamine Exposure in the Rat Nucleus Accumbens

**DOI:** 10.1523/ENEURO.0419-25.2025

**Published:** 2026-01-15

**Authors:** Samantha K. Saland, Florian Duclot, Mary K. Lobo, Mohamed Kabbaj

**Affiliations:** ^1^Department of Biomedical Sciences, College of Medicine, Florida State University, Tallahassee, Florida 32306; ^2^Program in Neuroscience, College of Medicine, Florida State University, Tallahassee, Florida 32306; ^3^Department of Neurobiology, School of Medicine, University of Maryland, Baltimore, Baltimore, Maryland 21201

**Keywords:** estrous cycle, ketamine, medium spiny neurons, nucleus accumbens, sensitization, translatome

## Abstract

The growing therapeutic promise of repeated, low-dose ketamine treatment across various psychopathologies—including depression and drug addiction—warrants clarity on its potential addictive properties and their associated mechanisms in both sexes. Accordingly, the present work examined the effects of intermittent low-dose ketamine in male and female rats on behavioral sensitization to the locomotor-activating effects of ketamine, as well as associated molecular profiles in dopamine D1- and D2-receptor-expressing medium spiny neurons (D1- and D2-MSNs) of the nucleus accumbens (NAc). Following intra-NAc infusion of a Cre-inducible RiboTag virus, locomotor activity was measured in adult Drd1a-iCre and Drd2-iCre male and female rats in either diestrus or proestrus following repeated administration of ketamine (0, 10, or 20 mg/kg, i.p.) to evaluate the development of locomotor sensitization. Female—but not male—rats developed sensitization to the locomotor-activating effects of ketamine, occurring more rapidly in proestrus than in diestrus females at the lower dose tested. To examine enduring context- and cell-type-specific changes in translating mRNAs associated with sensitization to ketamine, RNA sequencing was performed on polyribosome-bound mRNA of D1- and D2-MSNs isolated from the NAc of sensitized females in a drug-free state. A greater number of differentially expressed genes were observed selectively in D1-MSNs of ketamine-treated proestrus versus diestrus females, which were broadly related to regulation of transcription and epitranscriptional modification. These findings provide novel evidence of cell-type-specific and estrous cycle-dependent molecular profiles responsive to intermittent ketamine treatment in female rats and identify posttranscriptional mechanisms with relevance to ketamine's effects on behavioral plasticity.

## Significance Statement

Repeated low-dose ketamine treatment is often required to maintain its antidepressant efficacy. Given its history of recreational misuse, there is a need to identify predictors and neural signatures of susceptibility to ketamine's addictive liability using clinically relevant treatment regimens. Using a RiboTag viral vector approach in rats, we demonstrate that estrous cycle regulates both sensitized behavioral response to intermittent ketamine and enduring sensitization-associated posttranscriptional neuroadaptations in D1-receptor-expressing medium spiny neurons (MSNs) of the nucleus accumbens—a hub for reward and reinforcement. These changes occurred on a background of cycle stage-specific MSN subtype translatomes, providing some basis for differential treatment response within these cell populations and insights into the interaction between estrous cycle and ketamine's effects on brain plasticity.

## Introduction

A new era of therapeutic development for the treatment of depression and other neuropsychiatric disorders was ushered in upon discovery of ketamine's rapid therapeutic effects in depressed individuals (Berman et al., 2000; Zarate et al., 2006). Since this time, ketamine has been explored for off-label use in the treatment of a host of neuropsychiatric disorders (Glue et al., 2017; Martinotti et al., 2021), including substance abuse (Jones et al., 2018). Notwithstanding the clear therapeutic promise of this drug, repeated treatments are often required to achieve longer-lasting antidepressant response outcomes (Aan Het Rot et al., 2010; Murrough et al., 2013; Shiroma et al., 2014). Given its history of recreational misuse (Kalsi et al., 2011; Sassano-Higgins et al., 2016) and occurrence of abuse-related cognitive and affective deficits (Morgan et al., 2009, 2010; Chang et al., 2016), work is needed to better understand the potential abuse liability of ketamine as used for clinical symptom management.

Although clinical studies evaluating indicants of abuse liability of ketamine treatment in depressed patients are relatively sparse (Le et al., 2022), an abundance of preclinical evidence supports its addictive potential (De Luca and Badiani, 2011; Wright et al., 2017; Strong et al., 2019; Hagarty et al., 2024)—effects which are sex-, dose-, and context-dependent. Furthermore, our previous work identified estrous cycle as a moderator of ketamine's reinforcing properties in rats, where females in proestrus, but not diestrus, acquired and maintained ketamine self-administration (Wright et al., 2017). Though cycle stage-dependent differences in signaling pathway activation in some limbic structures (e.g., hippocampus) have been observed following acute low-dose ketamine in proestrus and diestrus female rats (Saland et al., 2022)—which typically reflect high and low levels of ovarian-derived hormones, respectively—it is unclear whether such neuroadaptive differences are apparent following intermittent treatment during these stages and to what extent they may be associated with the induction of behavioral plasticity following repeated drug exposures. As ketamine regimens for antidepressant use typically involve intermittent clinician-administered treatments, locomotor sensitization testing is one straightforward means of assessing early behavioral and molecular adaptations to repeated ketamine in rodents using treatment protocols that more closely resemble noncontingent clinical regimens. Furthermore, this drug-adaptive behavioral plasticity is linked with future motivational reward salience and reinstatement of drug-seeking behavior (Wise and Bozarth, 1987; Robinson and Berridge, 1993; Steketee and Kalivas, 2011), serving as an indirect indicant of potential abuse liability. Using this behavioral paradigm, the preclinical sex and/or estrous cycle differences observed in behavioral responsiveness to ketamine can be exploited to uncover neurobiological factors that may confer greater sensitivity to the development of such behavioral vulnerabilities across repeated treatments.

To this end, the nucleus accumbens (NAc) represents a central hub for reward processing and motivated behaviors whose disrupted functioning is strongly implicated in both depression and addiction (Xu et al., 2020; Ding et al., 2022). Several lines of evidence have associated structural and functional changes in the NAc with clinical improvement in depressive symptoms following ketamine (Abdallah et al., 2017; Chen et al., 2019; Siegel et al., 2021; Taraku et al., 2024). Moreover, sex- and dose-dependent changes in structural plasticity (Strong et al., 2017) and neuronal activation (Hagarty et al., 2024) in the NAc have been associated with addictive-like behavioral outcomes following repeated ketamine treatment. This region is primarily comprised of GABAergic medium spiny neurons characterized by their expression of either dopamine D1 or D2 receptors (D1- or D2-MSNs, respectively). Separable functional roles of these two primary NAc subpopulations in behavioral responses to addictive substances are well established, mediated in part through distinct patterns of signaling and transcriptional regulation (Lee et al., 2006; Bertran-Gonzalez et al., 2008; Kravitz et al., 2012; Lobo et al., 2013; Chandra et al., 2017; Fox et al., 2023). However, the extent to which these subtypes may be differentially affected by repeated ketamine treatment remains unclear.

Given the sex-dependent behavioral sensitivity to repeated ketamine administration in rodents and the involvement of the NAc in ketamine-associated behavioral outcomes, we employed a RiboTag viral vector approach (Sanz et al., 2015) in transgenic Drd1a-iCre and Drd2-iCre male rats and female rats in either proestrus or diestrus to evaluate the roles of sex and estrous cycle in early drug-adaptive neuroadaptations in D1- and D2-MSNs associated with the induction of locomotor sensitization to repeated intermittent ketamine. Using a noncontingent treatment protocol similar to that used clinically for depression, we show that sex and estrous cycle influence the occurrence and rate of development of locomotor sensitization to ketamine, respectively, that parallel enduring estrous cycle-specific and context-associated changes in NAc D1- but not D2-MSN translatomes in sensitized female rats.

## Materials and Methods

### Animals and housing

Adult male and female transgenic Long–Evans Drd1a-iCre [D1-Cre; LE-Tg(Drd1a-iCre)3Ottc] and Drd2-iCre [D2-Cre; LE-Tg(Drd2-iCre)1Ottc] rats bred in-house were used for experiments. Transgenic rats from both lines were obtained from the National Institute on Drug Abuse (NIDA transgenic rat project; Rat Resource and Research Center) and crossed with wild-type Long–Evans rats (Charles River Laboratories) as previously described (Martin et al., 2019; Pardo-Garcia et al., 2019; Strong et al., 2020). Heterozygous male and female D1-Cre and D2-Cre rats were selected following genotyping as previously described (Strong et al., 2020) and maintained in a breeding colony in a temperature- and humidity-controlled room under a 12:12 h light/dark cycle (lights on at 0800) until commencement of experimental procedures.

Adult D1-Cre and D2-Cre male (250–315 g) and female (180–225 g) rats used for experiments were pair-housed in 43 × 21.5 × 25.5 cm plastic cages and maintained on a 12 h:12 h reverse light/dark cycle (lights off at 0800) in a temperature- and humidity-controlled room. Food and water were available *ad libitum* for all animals throughout the duration of the study. A total of 94 male rats (46 D1-Cre, 48 D2-Cre) and 117 female rats (55 D1-Cre, 62 D2-Cre) were used in the present work. Of these rats, two males (D1-Cre) were removed from the study via termination due to a >15% reduction in body weight from unknown illness, and one additional D1-Cre male was identified as a statistical outlier during analysis of behavioral data and was therefore excluded (see below, Statistical analyses, for details). Furthermore, three females (two D1-Cre, one D2-Cre) were excluded from the study due to abnormal (>5 d) estrous cycles. All animal protocols were carried out in accordance with the NIH Guide for Care and Use of Laboratory Animals and approved by the Institutional Animal Care and Use Committee of Florida State University.

### Handling and estrous cycle monitoring

Male and female rats were habituated to basic handling procedures for 1 week prior to the commencement of behavioral procedures. Following habituation, daily estrous cycle monitoring and stage assignment of intact female rats were performed via vaginal lavage and characterization of cytologic smears as previously detailed (Saland et al., 2016, 2022; Saland and Kabbaj, 2018). In the current study, females were used in either the diestrus or proestrus stage of the estrous cycle—only rats exhibiting at least two consecutive 4 or 5 d cycles were included. Furthermore, postmortem smears were also collected to confirm staging assignments. Male rats received a similar brief daily handling to minimize potential confounds of handling and/or stress.

### Locomotor novelty response

Given that locomotor response to a novel environment can predict behavioral response to addictive substances (Hooks et al., 1992; Pierre and Vezina, 1997; Kabbaj and Akil, 2001), all rats were subjected to a 1 h novelty-induced locomotor test in circular activity chambers (Med Associates) prior to experimental testing as previously described (Kabbaj, 2006; Hollis et al., 2011). Locomotor activity was determined by crossings across four equidistant photo-beam sensors using an in-house software, where activity counts reflect the total number of beam breaks within a given session. Rats were categorized into low or high responders based on whether locomotor scores were situated below or above the median score, respectively, across all rats within each sex. Rats were assigned to experimental groups to achieve an equal representation of high and low responders across conditions, in addition to consideration of genotype and litter as factors.

### Stereotaxic surgery for viral delivery

Five days prior to the beginning of behavioral assessment, the RiboTag viral vector (AAV9-DIO-Rpl22-3xHA-Ires-EYFP, 1.4 × 10^12^ GC/ml; University of Maryland School of Medicine, Viral Vector Core) was delivered bilaterally into the NAc at the following coordinates: A/P = +1.7 mm (from bregma), M/L = ±1.4 mm, and D/V = −7.65 to −7.25 mm (from the skull surface) in three steps to span both the NAc shell and core regions. For all surgeries, rats were anesthetized with isoflurane gas (Covetrus) at a concentration of 5% for induction and 3–4% for maintenance at an oxygen flow rate of 1 L/min. A total of 1.2 µl of viral construct was delivered at a rate of 0.2 µl/min via bilateral cannula, which remained in place for 5 min following infusion to permit sufficient diffusion. Rats were administered carprofen (5 mg/kg, s.c.; Covetrus) prior to the first incision and a bupivacaine (0.25%) post-incision splash block as analgesics prior to surgery. Topical antibiotics were applied to the incision site during recovery to assist with healing.

### Ketamine treatment and locomotor activity assessment

**Figure 1.**
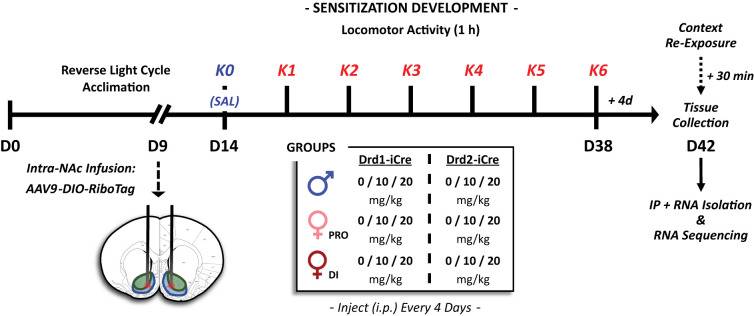
Experimental timeline depicting treatment and locomotor activity assessment schedules. NAc, nucleus accumbens; K, ketamine; IP, immunoprecipitation, PRO, proestrus; DI, diestrus.

The complete experimental design is presented in Figure 1. All drug administration and locomotor activity assessments occurred during the dark cycle in the behavioral testing context following a 30 min habituation period. All groups of D1-Cre and D2-Cre rats first underwent a 1 h test session in circular chambers immediately following a single injection of 0.9% saline (i.p.), where locomotor activity was recorded as described above. Then, every fourth day thereafter, separate groups of male rats and female rats in either proestrus or diestrus received either 0.0 mg/kg (*n* = 26 male, 21 proestrus, 23 diestrus), 10.0 mg/kg (*n* = 45 male, 21 proestrus, 21 diestrus), or 20.0 mg/kg (*n* = 20 male, 14 proestrus, 14 diestrus) racemic ketamine hydrochloride (1 ml/kg volume, i.p.; Dechra) and locomotor activity was immediately recorded for 1 h. A total of six ketamine treatments were administered. On occasion, females exhibiting 5 d cycles were instead injected every fifth day in the appropriate cycle stage. Four days following the final treatment session, rats were placed back into the behavioral testing environment and terminated 30 min later under nonstressful conditions (Fig. 1). Brains were quickly extracted, snap-frozen in 2-methylbutane at −25°C, and stored at −80°C until further processing.

### Polyribosome immunoprecipitation and RNA extraction from MSN subtypes

Tissue punches (2 mm) collected from 200 µm sections of the NAc of individual rats were used for subsequent processing. At least three separate litters from distinct breeding pairs within each treatment group were represented in the tissue used for immunoprecipitation (IP) in D1-Cre and D2-Cre proestrus and diestrus female rats to minimize potential litter bias. Furthermore, of the ketamine-treated females, only rats exhibiting >20% increase in activity levels over the course of treatment were included to increase the likelihood of enriching for translatomic changes relevant to the induction of behavioral plasticity observed in females following repeated treatment. Within these populations, the tissue was selected for processing within each ketamine group to represent the range of variability observed in percent increase in activity from the first to sixth treatment sessions while still controlling for litter bias (Proestrus D1, 25–106%; Proestrus D2, 31–92%; Diestrus D1, 21–85%; Diestrus D2, 24–141% increase). IP of polyribosomes from D1- and D2-MSNs was performed as previously described (Sanz et al., 2009, 2019; Chandra et al., 2015) with minor modifications. Frozen bilateral tissue punches were homogenized by douncing in a homogenization buffer (2.5–4% w/v) containing 50 mM Tris–HCl, pH 7.4, 100 mM KCl, 12 mM MgCl_2_, and 1% NP-40 substitute supplemented with 1 mM DTT, 100 µg/ml cycloheximide, 200 U/ml RNasin Plus (Promega), 1 mg/ml heparin, and EDTA-free protease inhibitor (ApexBio). Homogenate was spun at 10,000 × *g* at 4°C for 10 min and cleared lysate transferred to new tubes. Approximately 6–8% of lysate was retained as input and frozen at −80°C until further processing. The remaining lysate was incubated overnight at 4°C with an anti-HA antibody (BioLegend, Anti-HA.11; 1:200 v/v) with gentle end-over-end rotation, followed by an overnight incubation at 4°C with magnetic beads (Pierce; Protein A/G). The following day, beads were washed at 4°C three times with a high-salt buffer containing 50 mM Tris–HCl, pH 7.4, 300 mM KCl, 12 mM MgCl_2_, 1% NP-40 substitute, 0.5 mM DTT, and 100 µg/ml cycloheximide. Finally, genomic DNA removal and RNA purification were carried out using the RNeasy Plus Micro Kit (QIAGEN) according to the manufacturer's protocol. RNA was quantified using a Qubit Fluorometer (Qubit RNA HS Assay Kit; Thermo Fisher Scientific), and integrity was determined via TapeStation High Sensitivity ScreenTape analysis (Agilent). RNA integrity number equivalent values for all IP and input samples ranged from 8.2 to 9.4 (median 8.9).

### Real-time semiquantitative reverse transcription polymerase chain reaction (qRT-PCR)

RNA from separate samples of D1-Cre (*n* = 3) and D2-Cre (*n* = 2) rats infused intra-NAc with the RiboTag viral vector was prepared following procedures described above to validate enrichment of cell-type-specific polyribosome-bound transcripts from NAc tissue punches. One hundred nanograms of cDNA were synthesized using the iScript cDNA Synthesis Kit (Bio-Rad Laboratories) following the manufacturer's protocol. Relative mRNA expression was determined by qRT-PCR using iQ SYBR GREEN supermix (Bio-Rad Laboratories) as previously described (Hollis et al., 2011). Biological replicates were run in triplicate and quantified using the ΔΔC_T_ method (Livak and Schmittgen, 2001) using *Actb* as a reference gene. Primer sequences for D1-MSN- and D2-MSN-enriched genes (Gerfen et al., 1990; Schiffmann and Vanderhaeghen, 1993; Ince et al., 1997; Lobo et al., 2006) are presented as follows: *Drd1* (NM_012546.3) forward, CCAGCGGAGAGGGATTTCTC; reverse, AGGTGTCGAAACCGGATGAC; *Pdyn* (NM_019374.3) forward, CCATCAACCCCCTGATTTGC; reverse, TTGGTCAGTTCCGTGTAGCC; *Drd2* (NM_012547.3) forward, CTCAGGAGCTGGAAATGGAG; reverse, AGAGGACTGGTGGGATGTTG; *Adora2a* (NM_001357942.1) forward, ATTCCACTCCGGTACAATGG; reverse, AGTTGTTCCAGCCCAGCAT; and *Actb* (NM_031144.3) forward, AGTTCGCCATGGATGACGATAT; reverse, ATACCCACCATCACACCCTGG.

### Library preparation and RNA sequencing

IP and corresponding input RNA samples were sent to the FSU NGS Library Facility for the preparation of 80 barcoded and stranded RNA–seq libraries: *n* = 5 rats per group with eight groups (D1/D2-Cre × Diestrus/Proestrus × SAL/KET × IP/Input). All libraries were then pooled and sequenced on a single S1 flowcell (2 × 50 bp, NovaSeq 6000) at the Translational Sciences Laboratory at FSU. A total of 2,173 M paired-end raw reads (passing filter, >Q30, and demultiplexed) were generated (median, 27.56 M/sample). The data discussed in this publication have been deposited in NCBI's Gene Expression Omnibus (Edgar, 2002) and are accessible through GEO Series accession number GSE303789.

### Data processing, differential expression, and functional enrichment analyses

Raw reads were first processed for quality filtering and adapter trimming with fastp (v0.23.4; Chen, 2023), followed by verification of good quality using FastQC (v0.12.1; Andrews et al., 2015) before pseudoalignment to the rat transcriptome (mRatBN7.2, Ensembl annotations release 110) and quantification with Salmon (v1.10.2; Patro et al., 2017) using 1,000 bootstraps and the –validateMappings, –rangeFactorizationBins 4, –seqBias, –gcBias, and –recoverOrphans flags, to improve the sensitivity and specificity of mapping as well as correcting for common systematic biases (Love et al., 2016; Patro et al., 2017). Quantifications were thus summarized at the gene level using the R package tximport (v1.30.0; Soneson et al., 2016) and then processed for differential expression analysis using DESeq2 (v1.42.1; Love et al., 2014) using the following designs: ∼cycle (proestrus/diestrus) + genotype for genotype effect (D1-Cre vs D2-Cre) and ∼dose (SAL/KET) for treatment effect. In each analysis, surrogate variables were estimated using the sva package (v3.50.0; Leek et al., 2025) and then included in the design formula to remove hidden batch effects, as described by the DESeq2 authors. Library normalization, estimate of dispersion, count outlier detection and exclusion, and statistical testing were then conducted using DESeq2's default settings. Genes with a false discovery rate of <10% were classified as differentially expressed (DE); no threshold based on fold change was used. As per the authors’ recommendation, an initial inspection of all samples by principal component analysis revealed the presence of two outliers (1 IP D2-Cre Proestrus KET and 1 Input D2 Diestrus KET) likely resulting from technical processing given all animals used for RNA sequencing were chosen as representative of their respective group. These two outliers were thus excluded from the dataset before all statistical analyses of sequencing data.

The enrichment of gene ontologies and Kyoto Encyclopedia of Genes and Genomes (KEGG) pathways was tested using the Bioconductor package clusterProfiler (v4.10.1; Huber et al., 2015; Wu et al., 2021) and the full list of genes detected in our dataset as background. The redundancy of enriched gene ontology terms was then reduced to improve clarity by keeping one representative term among semantically similar terms using GOSemSim (Yu et al., 2010) as recommended by the authors. To provide an integrated view across multiple domains, we analyzed the functional enrichment in differentially expressed genes (DEGs) in ketamine-treated proestrus females using Enrichr-KG (Evangelista et al., 2023) to return the top five enriched terms from each of the Reactome 2022, WikiPathways 2021_Human, KEGG_2021_Human, MGI_Mammalian_Phenotype_Level_4_2021, and GO Biological Process 2021 databases. Enrichr-KG results were then depicted as a network using Cytoscape (Shannon et al., 2003).

### Immunohistochemistry (IHC) and image acquisition

To validate the RiboTag viral expression and placement accuracy, we anesthetized D1-Cre and D2-Cre rats that received intra-NAc infusions 3 weeks later with EUTHASOL (Virbac) and transcardially perfused first with 0.2 M phosphate-buffered saline (PBS), followed by 4% paraformaldehyde (PFA) in PBS. Brains were extracted and postfixed at 4°C in 4% PFA overnight, then washed three times with PBS and stored in PBS with 0.1% sodium azide at 4°C. Brains were sectioned using a vibratome (Leica, VT1200S) at a thickness of 40 µm, and NAc sections of D1- and D2-Cre rats containing EYFP reporter expression were selected for IHC use. The tissue was washed three times with PBS and then permeabilized for 1 h at room temperature (RT) in PBS containing 0.3% Triton X-100. Sections were blocked for 1 h (RT) in 0.3% Triton X-100 in PBS with 5% normal goat serum (NGS), then incubated for 48 h at 4°C in primary antibody (chicken anti-GFP, 1:500 v/v; Abcam #ab13970) with 5% NGS. The tissue was washed five times for 5 min each in 0.3% Triton X-100 PBS and then incubated overnight at 4°C with secondary antibody (anti-chicken Alexa Fluor 488, 1:1,000 v/v; Invitrogen #A11039). The following day sections were washed, slide-mounted, dried, and coverslipped using Fluoroshield mounting medium with DAPI (Sigma-Aldrich) prior to image processing. Representative images demonstrating viral placement and reporter expression were generated using a Keyence BZ-X710 at 4× magnification.

### Statistical analysis

All behavioral data were analyzed either by GraphPad Prism (version 10; GraphPad Software) or the R (v4.3.1) package rstatix (Kassambara, 2023). Locomotor activity was first analyzed by repeated-measure three–way analysis of variance (ANOVA), with dose and sex/cycle (male, proestrus, diestrus) as between-subject factors and session (day of testing) as the within-subject factor. Pending significant main effects or interactions, data were then analyzed using repeated-measure two–way ANOVAs with the Geisser–Greenhouse correction for sphericity applied. Ketamine dose served as the between-subject factor and session as a within-subject factor, using Šídák's multiple-comparison post hoc tests. Two-way ANOVAs were performed to compare acute response (Session 1) between groups based on sex/cycle or dose with both dose and cycle as between-subject factors, followed by Tukey's multiple-comparison post hoc tests. Time-binned data were analyzed by repeated-measure four–way ANOVA, where cycle and dose served as between-subject factors and session and time bin as within-subject factors. Subsequent two-way ANOVAs and post hoc testing were performed as described above. Partial eta squared and common omega squared were calculated as measures of the effect size in three-/four-way ANOVAs and two-way ANOVAs, respectively. mRNA levels assessed via qRT-PCR were subjected to two-tailed unpaired *t* tests. The probability of occurrence by chance alone of the overlap between two sets of genes was tested using a hypergeometric test from the R stats package (R Core Team, 2024). Data are presented as means ± SEMs and alpha set to 0.05 for all statistical analyses.

## Results

### Sex-dependent sensitivity to the locomotor-activating effects of ketamine

**Figure 2.**
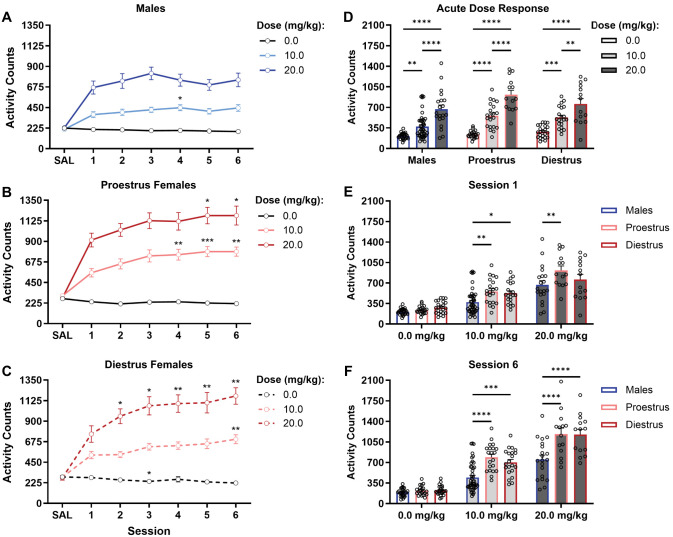
Sex-dependent sensitivity to the locomotor-activating effects of intermittent ketamine. ***A–C***, Activity counts in males (panel ***A***; *n* = 26 0.0 mg/kg; *n* = 45 10.0 mg/kg; *n* = 20 20 mg/kg), proestrus females (panel ***B***; *n* = 21 0.0 mg/kg; *n* = 21 10.0 mg/kg; *n* = 14 20.0 mg/kg), and diestrus females (panel ***C***; *n* = 23 0.0 mg/kg; *n* = 21 10.0 mg/kg; *n* = 14 20.0 mg/kg) across the six intermittent ketamine injections show development of sensitization to ketamine in females—but not males—at 10.0 and 20.0 mg/kg doses. ***D***, Dose-response comparisons within male and female groups of rats show similar dose-dependent increases in activity following a single ketamine injection. ***E***, ***F***, Within-dose activity comparisons after the first (panel ***E***) and sixth (panel ***F***) injections demonstrate greater locomotor stimulant response in females compared with males following ketamine injections yet similar baseline activity after vehicle administration. Data are depicted as means ± SEMs; (***A–C***) *p < 0.05; **p < 0.005; ***p < 0.0005 versus first injection using Šídák's post hoc; (***D***) ***p = 0.0003 versus 0.0 mg/kg (diestrus); **p < 0.005; ****p < 0.0001 versus 0.0 or 10.0 mg/kg ketamine using Tukey's post hoc. ***E***, ***F***, *p = 0.0135; **p < 0.003; ***p = 0.0001; ****p < 0.0001 versus males using Tukey's post hoc. All statistical analyses corresponding to this figure are presented in Extended Data Figure 2-1.

We first investigated whether estrous cycle and sex influenced the locomotor-activating effects of repeated intermittent ketamine administered once every 4–5 d, when females were in either proestrus or diestrus (see Fig. 1 for detailed experimental timeline). Consistent with previous work from our lab (Strong et al., 2017; Schoepfer et al., 2019), both sex (*F*_(2,196)_ = 27.28; *p* < 0.0001) and dose (*F*_(2,196)_ = 207.18; *p* < 0.0001; sex × dose, *F*_(4,196)_ = 4.911; *p* = 0.0009) significantly affected locomotor response to ketamine (Fig. 2). Under this treatment regimen, any increases observed across the six treatment sessions (*F*_(4.16,814.47)_ = 28.94; *p* < 0.0001) were both sex-/cycle- (sex/cycle × session, *F*_(8.31,814.47)_ = 4.674; *p* < 0.0001) and dose-dependent (dose × session, *F*_(8.31,814.47)_ = 11.886; *p* < 0.0001; sex × dose × session, *F*_(16.62,814.47)_ = 2.115; *p* = 0.006).

10.1523/ENEURO.0419-25.2025.f2-1 Figure 2-1 Behavioral statistical analysis tables. Download Figure 2-1, XLS file.

While males did exhibit dose-dependent increases in locomotor activity throughout the treatment period (Fig. 2*A*; dose, *F*_(2,88)_ = 53.20; *p* < 0.0001; session, *F*_(3.683,324.1)_ = 2.606; *p* = 0.0402), no apparent sensitization developed at any dose by the sixth treatment (vs Session 1), apart from a modest increase in males following the fourth injection of 10 mg/kg ketamine (*p* = 0.0478) that was not sustained (Extended Data Fig. 2-1). In contrast, locomotor activity significantly increased across the six sessions after treatment with ketamine in both proestrus (Fig. 2*B*; session, *F*_(3.328,176.4)_ = 19.02; *p* < 0.0001) and diestrus (Fig. 2*C*; session, *F*_(3.595,197.7)_ = 17.28; *p* < 0.0001) females in a dose-dependent manner (proestrus: dose, *F*_(2,53)_ = 74.77; *p* < 0.0001; dose × session, *F*_(10,265)_ = 5.368; *p* < 0.0001; diestrus: dose, *F*_(2,55)_ = 72.45; *p* < 0.0001; dose × session, *F*_(10,275)_ = 9.682; *p* < 0.0001). Although both proestrus and diestrus female rats sensitized to ketamine at the doses tested, estrous cycle differences were apparent in the rate at which sensitization developed. This was first evident at the lower dose administered (10 mg/kg), where activity levels significantly increased and persisted in proestrus females with just four treatments (*p* = 0.0069 vs Session 1; Fig. 2*B*), but not until the sixth and final treatment (*p* = 0.0068 vs Session 1; Fig. 2*C*) in diestrus rats. Furthermore, the magnitude of increase in activity levels within-subject from the first treatment to the final session (six) was greater in proestrus females (55.01 ± 12.50% increase) compared with diestrus females (40.38 ± 10.64% increase). In contrast, an enhanced locomotor response to 20 mg/kg occurred rapidly after the second treatment in diestrus females (*p* = 0.0285 vs Session 1) but only after five doses in proestrus females. However, as activity levels were similar between these groups during the sixth session, this discrepancy may in part reflect the greater acute response observed at this dose in proestrus versus diestrus females (see below) at 20 mg/kg—a difference not observed at the lower dose (see below).

Despite clear sex differences in the susceptibility to develop locomotor sensitization to ketamine, all rats regardless of sex or cycle stage exhibited clear and dose-dependent (dose, *F*_(2,196)_ = 95.42; *p* < 0.0001) acute locomotor stimulant responses following the first injection of ketamine (Fig. 2*D*), with a strong effect of sex (sex/cycle, *F*_(2,196)_ = 9.849; *p* < 0.0001; interaction, *F*_(4,196)_ = 1.903; *p* = 0.1115) where females generally traveled greater distances than males (Fig. 2*E*). This sex difference was observed in both proestrus (*p* = 0.0025 vs males) and diestrus (*p* = 0.0135 vs males) females following 10 mg/kg ketamine, whereas only proestrus, but not diestrus, females exhibited an enhanced locomotor response to 20 mg/kg when compared with males (*p* = 0.0021), suggesting an enhanced acute locomotor response in proestrus females to the highest dose of ketamine used. This estrous cycle effect was absent by the sixth treatment session (Fig. 2*F*), with significantly greater activity counts in both groups of females receiving 10 mg/kg (proestrus, *p* < 0.0001; diestrus, *p* = 0.0001) and 20 mg/kg (proestrus, diestrus, *p* < 0.0001) ketamine when compared with males (sex/cycle, *F*_(2,196)_ = 27.65; *p* < 0.0001; dose, *F*_(2,196)_ = 180.4; *p* < 0.0001; interaction, *F*_(4,196)_ = 5.638; *p* = 0.0003). These differences could not be accounted for by baseline differences in activity levels, which were similar between all saline-treated groups across sessions (*p*s > 0.05; Extended Data Fig. 2-1).

### Temporal patterns of locomotor response to ketamine

**Figure 3.**
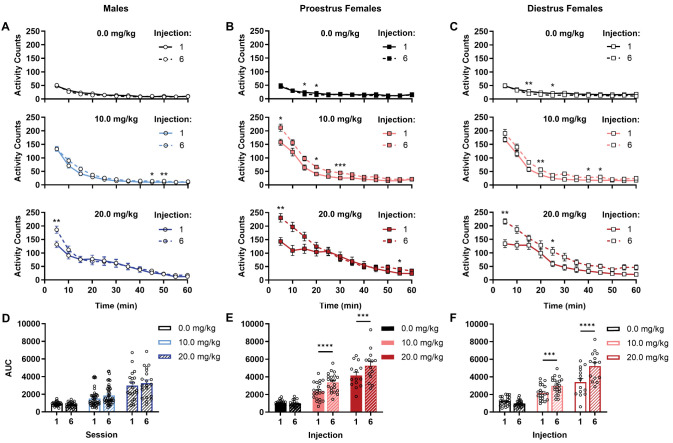
Temporal patterns of locomotor response to repeated ketamine across sex and estrous cycle. ***A***, Only males treated with 20.0 mg/kg ketamine exhibited increased locomotor activity 5 min after the sixth compared with first injection, with activity levels otherwise similar between first and last injections in all male treatment groups. ***B***, ***C***, Increased activity during the sixth compared with first sessions was most notable from 5 to 30 min post-treatment in proestrus (panel ***B***) and diestrus (panel ***C***) females treated with 10.0 mg/kg (middle) or 20.0 mg/kg (bottom) ketamine. ***D–F***, AUC values generated from time-binned activity plots confirm the absence of locomotor sensitization in males (panel ***D***) regardless of dose and sensitized locomotor response by the sixth injection in proestrus (panel ***E***) and diestrus (panel ***F***) females treated with either 10.0 or 20.0 mg/kg ketamine. Data are expressed as means ± SEMs; *p < 0.05; **p < 0.01; ***p < 0.001; ****p < 0.0001 versus Injection/Session 1 using Šídák's post hoc. All statistical analyses corresponding to this figure are presented in Extended Data Figure 2-1. Between-dose comparisons of time-binned activity from sessions 1 and 6 are shown in Extended Data Figure 3-1.

To assess activity patterns throughout the hour-long sessions after ketamine administration, we generated time-course profiles following the first and sixth treatments in males (Fig. 3*A*), proestrus females (Fig. 3*B*), and diestrus females (Fig. 3*C*). As the development of sensitization was determined by a significant increase in activity from session 1 to session 6, area-under-the-curve (AUC) values were then generated from these response profiles and compared between treatment sessions 1 and 6 across sex, cycle, and treatment groups (Fig. 3*D*–*F*) as a secondary means of confirmation of sensitized response to ketamine (or lack thereof) in male and female rats. Consistent with cumulative activity counts, main effects of sex/cycle (*F*_(2,196)_ = 23.03; *p* < 0.0001), dose (*F*_(2,196)_ = 174.80; *p* < 0.0001), and session (*F*_(1,196)_ = 70.19; *p* < 0.0001) were observed, with significant interactions supporting cycle- (sex/cycle × session × time bin, *F*_(5.92,580.55)_ = 2.34; *p* = 0.0310) and dose-dependent (dose × session × time bin, *F*_(5.92,580.55)_ = 6.691; *p* < 0.0001; sex/cycle × dose × time bin, *F*_(10.13,496.53)_ = 3.446; *p* = 0.0002) effects on temporal response patterns between the first and sixth sessions. A full detailing of statistical analyses can be found in Extended Data Figure 2-1. In general, activity levels were similar throughout the course of treatment in all saline-treated rats (Fig. 3*A–C*, top; all AUC *p*s > 0.05; 0.0 mg/kg Injection 6 vs 1; Extended Data Fig. 2-1), confirming that repeated saline injections alone did not sensitize locomotor response.

10.1523/ENEURO.0419-25.2025.f3-1 Figure 3-1 Dose comparison of temporal locomotor response patterns for first and last ketamine treatments within sex and estrous cycle stage. ***A***, Males (top), proestrus females (middle), and diestrus females (bottom) exhibit dose-dependent increases in activity most notably during the first 25 min after a single 10.0 (light blue, pink asterisks) or 20.0 (dark blue, red asterisks) mg/kg ketamine injection compared to vehicle. ***B***, Between-dose comparisons after the sixth ketamine injection in males (top), proestrus females (middle) and diestrus females (bottom) demonstrate extended increases in activity compared to saline across the hour-long session. Data are expressed as means ± SEMs; *p < 0.05, **p < 0.01, ***p < 0.001, ****p < 0.0001 vs. 0.0 mg/kg; ^#^p < 0.05, ^##^p < 0.01, ^###^p < 0.001, ^####^p < 0.0001, 20.0 mg/kg vs. 10.0 mg/kg; Tukey’s multiple comparisons. Download Figure 3-1, TIF file.

Apart from a short-lived increase in locomotor response in males to repeated treatment with 20 mg/kg ketamine for the first 5 min following administration (Fig. 3*A*, bottom; *p* = 0.0038 vs Injection 1), no meaningful ketamine-induced increases in activity were observed throughout the hour-long sessions in males at any dose (Fig. 3*D*; dose, *F*_(2,88)_ = 35.10; *p* < 0.0001; injection, *F*_(1,88)_ = 2.280; *p* = 0.1356; interaction, *F*_(2,88)_ = 1.873; *p* = 0.1598). AUC values from temporal response patterns in proestrus (Fig. 3*E*; dose, *F*_(2,53)_ = 69.46; *p* < 0.0001; injection, *F*_(1,53)_ = 28.00; *p* < 0.0001; interaction, *F*_(2,53)_ = 10.10; *p* = 0.0002) and diestrus (Fig. 3*F*; dose, *F*_(2,55)_ = 59.34; *p* < 0.0001; injection, *F*_(1,55)_ = 39.94; *p* < 0.0001; interaction, *F*_(2,55)_ = 23.29; *p* < 0.0001) females confirm the enhanced locomotor response to repeated ketamine at both 10 mg/kg (Fig. 3*B*,*C*, middle; *p*s ≤ 0.0005 vs Injection 1) and 20 mg/kg (Fig. 3*B*,*C*, bottom; *p*s ≤ 0.0002 vs Injection 1). Time-binned data in 10 mg/kg-treated females revealed comparatively greater activity levels primarily over the first 30 min following the sixth treatment compared with the first (Fig. 3*B*,*C*, middle; *p < 0.05; **p = 0.0053; ***p = 0.0002), with more modest increases observed through the remainder of the hour-long sessions (*p*s < 0.04). Locomotor-activating effects of 20 mg/kg ketamine were most apparent the first 5 min after treatment in proestrus females (*p* = 0.0067), returning to the first-session levels shortly thereafter. In contrast, activity remained significantly elevated compared with the first treatment session throughout the first 25 min at this dose in diestrus females (*p = 0.0318; **p = 0.0088), with nonsignificant but meaningful (cumulatively) increases in activity counts over the remaining 35 min. Particularly in females, response profiles following the first treatment with 20 mg/kg exhibited blunted activity peaks at 5 min post-treatment near or slightly below those observed with 10 mg/kg. This was followed by a protracted period of locomotor stimulation which was longer in duration in proestrus females (40 min; *p* = 0.0391 vs saline; Extended Data Fig. 3-1) than in diestrus females (25 min; *p* = 0.0074 vs saline; Extended Data Fig. 3-1). It is notable that these blunted but extended response patterns were not observed at the sixth injection of 20 mg/kg (Extended Data Fig. 3-1), which followed a more typical dose-dependent peak at 5 min followed by gradual decreases in activity over the course of the session. While fine motor movements were not assessed here and this time-course analysis is descriptive, it is relevant that similar observations reported in ketamine-treated rats were associated with ataxic effects of higher doses over the first 15 min following treatment and a tolerance to those effects with repeated intermittent ketamine administration (Elersič et al., 2024, 2025).

### Generation of polyribosome-associated transcriptomes of NAc D1- and D2-MSNs in female rats

**Figure 4.**
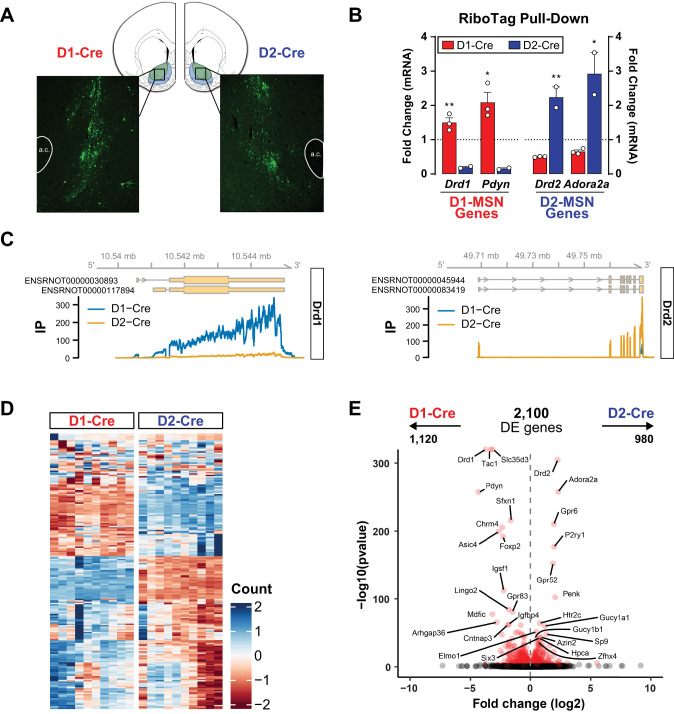
Validation of RiboTag viral expression and isolation of NAc D1- and D2-MSNs in female rats. ***A***, Representative image of EYFP (AAV-DIO-RiboTag) expression in the NAc of D1-Cre and D2-Cre rats. ***B***, Cell-type-specific enrichment of ribosome-associated mRNA for known MSN subtype genes. Data represent the fold change of IP relative to input samples isolated from the NAc of D1-Cre (*n* = 3) and D2-Cre (*n* = 2) rats, where all values were normalized to *Actb.* ***C***, Normalized read coverage of the *Drd1* and *Drd2* gene loci in IP samples from D1-Cre and D2-Cre rats. ***D***, Heatmap of the rlog-transformed read counts of the 200 genes with the most reads counts. Genes are depicted in rows, whereas samples (D1-Cre or D2-Cre IP) are depicted in columns. ***E***, A volcano plot depicting the log2 fold change (*x*-axis) against the −log10 of the uncorrected *p* value (*y*-axis) for each gene. Differentially expressed genes are shown in red, and the 15 genes with the lowest adjusted *p* values biased in D1- or D2-Cre IP samples are labeled. Data are depicted as means + SEMs; *p < 0.05; **p < 0.01 D2 versus D1, unpaired *t* test. a.c., anterior commissure. A full list of results for the differential expression analysis is available in Extended Data Figure 4-1.

Previous work has demonstrated that repeated ketamine induces alterations in structural plasticity within the NAc associated with locomotor sensitization following intermittent treatment in rats (Strong et al., 2017; Schoepfer et al., 2019). This increased locomotor response was associated with greater protein levels of ΔFosB within this region in males, but was not apparent in the NAc of sensitized females at lower doses (Strong et al., 2017; Schoepfer et al., 2019), suggesting that alternative mechanisms underlie the enhanced sensitivity of females to the locomotor-activating effects of repeated ketamine treatment. Given the differential roles of D1- and D2-expressing NAc MSNs in psychostimulant-induced behavioral responses (Lobo et al., 2010; Chandra et al., 2015; Kai et al., 2015; Van Zessen et al., 2021), we employed a RiboTag viral vector approach (Sanz et al., 2015, 2019) to isolate polyribosome-bound mRNA from D1- and D2-MSN-specific populations within the NAc of proestrus and diestrus females following repeated ketamine treatment. As the primary aim of this work was to relate early translatomic neuroadaptations in NAc MSNs following repeated ketamine to the induction of sensitized locomotor response, the tissue from male rats was not processed, as sensitization induction was not observed in males under the intermittent ketamine treatment protocol used in the present work. Confirmation of RiboTag viral expression and placement within the NAc are presented in Figure 4*A*, which shows robust expression of EYFP in the NAc of D1-Cre and D2-Cre transgenic rats that received bilateral injections of RiboTag AAV.

10.1523/ENEURO.0419-25.2025.f4-1 Figure 4-1 Differential expression analysis of genes isolated from D1- versus D2-MSNs in the nucleus accumbens of either all saline-treated females (combined), proestrus females only, or diestrus females only. Download Figure 4-1, XLS file.

In D1-Cre and D2-Cre rats infused with the RiboTag viral vector, Cre-dependent expression of ribosomal hemagglutinin (HA) tags permitted HA-antibody-facilitated IP of polyribosomes from which D1- and D2-MSN-specific mRNA could then be isolated. Enrichment of the D1-MSN genes, *Drd1* (*p* = 0.0053) and *Pdyn* (*p* = 0.0140), and the D2-MSN genes, *Drd2* (*p* = 0.0046) and *Adora2a* (*p* = 0.0161), in their respective cell types (as previously reported; Gerfen et al., 1990; Schiffmann and Vanderhaeghen, 1993; Ince et al., 1997; Lobo et al., 2006) were first demonstrated via qRT-PCR (Fig. 4*B*) and confirmed by greater RNAseq reads coverage of the *Drd1* gene by D1- than D2-Cre IP libraries and of the *Drd2* gene by D2- than D1-Cre IP libraries (Fig. 4*C*). The top 200 genes with the most read counts also show vast differences between D1- and D2-Cre samples, denoting a stark contrast in the gene expression profile between the two groups (Fig. 4*D*). Accordingly, 2,100 genes were DE between D1- and D2-Cre IP samples, with 1,120 (53%) and 980 (47%) being D1- and D2-biased, respectively (Fig. 4*E*; Extended Data Fig. 4-1). Among the most well known cell-type-specific DEGs were *Drd1*, *Pdyn*, *Tac1*, and *Chrm4* in D1-MSNs and *Drd2*, *Adora2a*, *Penk*, and *Gpr6* in D2-MSNs (Gerfen et al., 1990; Schiffmann and Vanderhaeghen, 1993; Ince et al., 1997; Lobo et al., 2006), validating the robust selective enrichment of these NAc MSN subtypes.

### Translatome profiles of D1- and D2-MSNs in proestrus and diestrus female rats

**Figure 5.**
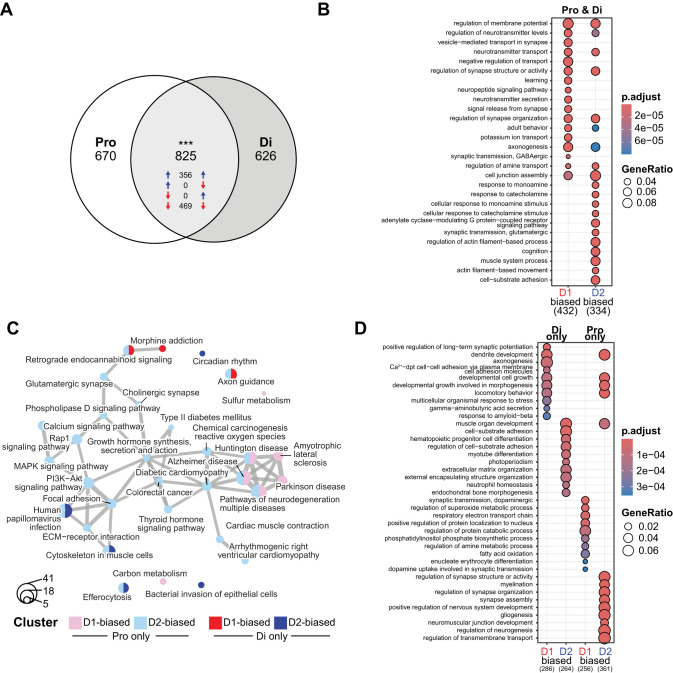
Estrous cycle influence on the cell-type-specific pattern of gene expression in the NAc at baseline. ***A***, The number of DEGs between D2- and D1-Cre IP samples common or distinct between proestrus (Pro) and diestrus (Di) is depicted. In the common genes, the direction of change was consistent between cycle stages: upregulated (D2-bias, blue arrows) or downregulated (D1-bias, red arrows). ***B***, The functional enrichments in the common set of genes (Pro & Di) reveal differences in the ontologies of the biological processes biased in each MSN subtype. ***C***, ***D***, The DEGs specific to each estrous cycle stage are enriched in distinct KEGG terms (panel ***C***) and biological process ontologies (panel ***D***), highlighting the uniqueness in subtype-specific regulations by the estrous cycle. In all panels, the circle size depicts the number of associated genes. ***p < 0.001, hypergeometric test for the overlap between two sets of genes. A full list of results for the differential expression analysis is available in Extended Data Figure 4-1. Functional enrichment analyses are reported for all comparisons related to this figure in Extended Data Figure 5-1.

Given the relevance of estrous cycle to behavioral outcomes following ketamine treatment in female rats, we first established ribosome-bound mRNA expression profiles in MSN subtypes separately for saline-treated proestrus and diestrus females to determine whether estrous cycle influences cell-type-specific expression patterns in the NAc at baseline (Fig. 5). A total of 825 genes were commonly DE between D1- and D2-MSNs in both estrous cycle stages (*p* < 0.001; Fig. 5*A*; Extended Data Fig. 4-1). The direction of change within these DEGs was the same between proestrus and diestrus females, supporting the independence of these MSN subtype differences from estrous cycle regulation during these two stages—469 were D1-biased, while 356 DEGs were D2-biased in their expression patterns. A predominant functional cluster of DEGs enriched in D1-MSNs related to neuronal communication through the regulated secretory pathway, demonstrating differential translation of genes involved in vesicle-mediated transport and neurotransmitter and neuropeptide secretion at the synapse in D1- compared with D2-MSNs (Fig. 5*B*), with many DEGs encoding synaptic vesicle- and neuronal dense core vesicle-localized proteins (Extended Data Fig. 5-1). Several D1-MSN-enriched genes were also related to transmembrane potassium ion transport, as well as GABAergic synaptic transmission. In contrast, D2-MSN DEGs were enriched in processes involved in glutamatergic synaptic transmission and G-protein-coupled receptor signaling—particularly in response to catecholamines (Fig. 5*B*). Notably, structural processes comprised of several extracellular matrix structural constituents (Extended Data Fig. 5-1) were highly represented among D2- versus D1-MSN–enriched genes, including regulation of actin filament-based processes and cell-substrate adhesion.

10.1523/ENEURO.0419-25.2025.f5-1 Figure 5-1 Functional enrichment analyses of genes differentially expressed in nucleus accumbens D1- versus D2-MSNs in either proestrus or diestrus female rats. Download Figure 5-1, XLS file.

Despite the strong concordance between both groups of females in D1- versus D2-MSN-enriched DEGs, large populations of estrous cycle-specific genes were differentially enriched in MSN subtypes exclusively in proestrus or diestrus rats (Fig. 5*A*). In proestrus females, 670 genes were DE between D1- and D2-MSNs, with an additional 626 identified only in diestrus females (Extended Data Fig. 4-1). An overlapping set of DEGs in proestrus-only D1-MSNs were broadly associated with neurodegenerative diseases, while those enriched in proestrus-only D2-MSNs were predominantly related to intracellular signaling pathways (PI3K-Akt, MAPK, and Rap1 signaling; Fig. 5*C*). On the other hand, diestrus-only D1-MSN DEGs were associated with morphine addiction, whereas focal adhesion was enriched in D2-MSN-biased genes. Interestingly, though axon guidance and retrograde endocannabinoid signaling KEGG pathways were significantly enriched in both proestrus-only and diestrus-only datasets, the direction of regulation differed depending on estrous cycle stage—D1-biased in diestrus only and D2-biased in proestrus only.

While both proestrus-only and diestrus-only DEGs exhibited similar enrichment in synaptic activity processes in D1-MSNs and structural processes in D2-MSNs, Figure 5*D* demonstrates the uniqueness in subtype-specific regulation of biological processes by the estrous cycle. For example, GABA secretion and synaptic vesicle/postsynaptic density localization were enriched in diestrus-only D1-MSNs, whereas D1-biased DEGs in proestrus females participate in dopaminergic transmission, transport, and uptake (Fig. 5*D*; Extended Data Fig. 5-1). In contrast, D2-biased diestrus-only DEGs were related to cell-substrate adhesion and extracellular matrix organization compared with synapse assembly, transmembrane transport, and cell junction assembly enrichment in proestrus-only D2-MSNs. The cell-type-specific regulation of differential translation in the NAc by the estrous cycle is further evidenced by the enrichment of distinct molecular functions in MSN subtypes in diestrus versus proestrus females (Extended Data Fig. 5-1).

### Estrous cycle influences translational regulation by repeated ketamine in NAc MSNs in a cell-type-specific manner

**Figure 6.**
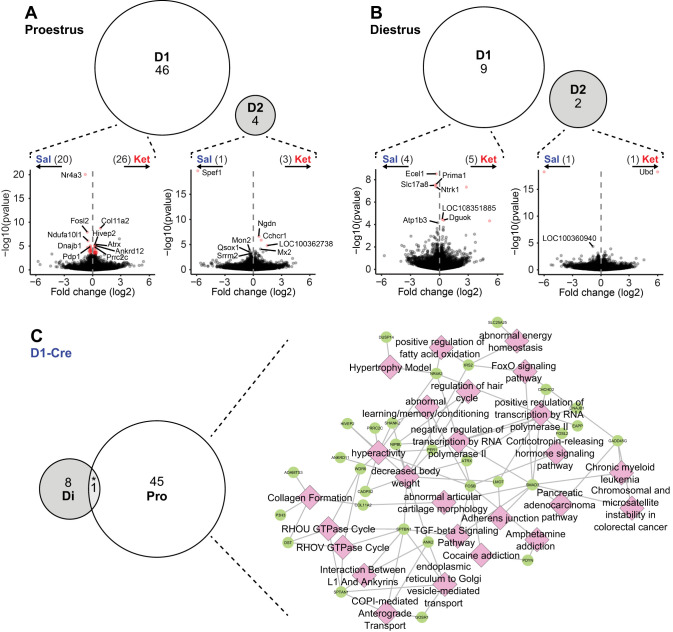
Estrous cycle influence on the cell-type-specific effects of ketamine. ***A***, ***B***, Intermittent ketamine treatment results in a greater number of DEGs in proestrus (Pro, panel ***A***) than diestrus female rats (Di, panel ***B***), and primarily involves D1-MSNs. Volcano plots (bottom) depict the log2 fold change (*x*-axis) against the −log10 of the uncorrected *p* value (*y*-axis) for each gene. Differentially expressed genes are shown in red, and up to five genes with the lowest adjusted *p* values up- or downregulated by ketamine are labeled. ***C***, Most genes regulated by ketamine in D1-Cre IP samples in proestrus are distinct from those affected in diestrus (left side); their functional enrichment is displayed on the right side, with enriched terms depicted as pink diamonds, and genes as green circles. *p < 0.05, hypergeometric test for the overlap between two sets of genes. All results from differential expression and functional enrichment analyses related to this figure are available in Extended Data Figures 6-1 and 6-2, respectively.

The behavioral data presented herein confirm the greater susceptibility of female rats to develop locomotor sensitization to intermittent ketamine compared with males. However, it remains unclear the extent and nature of molecular changes incurred that may confer this enhanced behavioral response to ketamine over time. Accordingly, translatomes of NAc D1- and D2-MSNs isolated from rats that developed clear locomotor sensitization to 10 mg/kg ketamine were compared against saline-treated controls following context re-exposure in a drug-free state. Proestrus and diestrus females were directly compared in order to determine whether the faster rate of sensitization in proestrus females reflected greater ketamine-induced changes in mRNA translation. Consistent with behavioral data, a greater number of DEGs were identified in proestrus compared with diestrus females, with 46 genes DE in D1-MSNs and 4 in D2-MSNs (Fig. 6*A*). Only nine DEGs were identified in diestrus D1-MSNs, with just two in D2-MSNs (Fig. 6*B*). Only one gene, *Ecel1*, was commonly regulated by ketamine treatment in D1-MSNs from proestrus and diestrus females (*p* = 0.0171; Fig. 6*C*) but in opposite directions—up in proestrus and down in diestrus. A full list of treatment-related DEGs by estrous cycle and MSN subtype is presented in Extended Data Figure 6-1.

10.1523/ENEURO.0419-25.2025.f6-1 Figure 6-1 Differential expression analysis for cell-type-specific effects of repeated ketamine treatment on gene expression in D1- and D2-MSNs in the nucleus accumbens of female rats treated either during proestrus or diestrus stages of the estrous cycle. Download Figure 6-1, CSV file.

10.1523/ENEURO.0419-25.2025.f6-2 Figure 6-2 Functional enrichment analysis of genes differentially expressed in D1-MSNs following repeated ketamine treatment in proestrus female rats. Download Figure 6-2, CSV file.

Figure 6*C* depicts enrichment of ketamine-regulated DEGs in D1-MSNs of proestrus females. Interestingly, of the 20 genes downregulated by repeated ketamine in proestrus D1-MSNs, 10 were immediate early genes (IEGs), including several transcription factors (*Fosb*, *Fosl2*, *Nr4a3*) known to be regulated by repeated psychostimulant (e.g., cocaine and amphetamine) exposure. Furthermore, 14 of those 20 downregulated DEGs were late response genes (LRGs), typically induced later on (∼4 h), secondary to IEGs, following neuronal depolarization in rat striatal neurons (Phillips et al., 2023a)—including the known D1-MSN-enriched genes *Tac1* and *Pdyn*. Proestrus-specific D1-MSN DEGs upregulated by ketamine were related to structure and function of the cell membrane, intracellular transport, transcription, and posttranscriptional regulation of mRNA (Fig. 6*C*; Extended Data Fig. 6-2). Genes encoding ankyrin, spectrin, and dystonin proteins (*Ank2*, *Sptan1*, *Sptbn1*, *Dst*) which play crucial roles in maintaining cytoskeletal organization and membrane trafficking were upregulated by ketamine, in addition to several genes involved in transcriptional regulation—*Atrx*, *Ankrd11*, *Ankrd12*, *Hivep2*, *Lmo7*, *Nipbl*, and *Smad3*. Importantly, numerous D1-MSN upregulated genes participate in N6-methyladenosine (m6A) RNA modification processes, including *Zc3h13*, *Rbm3*, *Prrc2c*, and *Smad3*—the latter of which is also involved in D1-receptor-dependent alternative splicing of *Fosb* mRNA following drug exposure (Krapacher et al., 2022).

Among the few DEGs upregulated by ketamine in diestrus females (Fig. 6*B*) was *Dguok*, an enzyme involved in mitochondrial DNA replication and repair. Downregulated D1-MSNs genes in this group include the vesicular glutamate transporter, *Slc17a8*, and two additional genes that contribute to cholinergic neuron survival and PI3K/Akt signaling (*Ntrk1*) and regulation of cholinergic synaptic activity (acetylcholinesterase membrane anchor, *Prima1*). Overall, the regulation of mRNA translation by repeated ketamine is much greater in D1-MSNs and is highly estrous cycle-dependent and mostly nonoverlapping between proestrus and diestrus females.

## Discussion

Here we show that both sex and estrous cycle influence the development of sensitization to the locomotor-activating effects of ketamine and provide the first evidence that estrous cycle regulates differential translation between NAc MSN subtypes at baseline and in response to intermittent ketamine. While increased activity with repeated treatments was not apparent in males, females sensitized to both 10 and 20 mg/kg ketamine regardless of cycle stage. However, the estrous cycle did influence the rate at which sensitization developed, occurring more rapidly during proestrus than diestrus at the lower dose tested. Estrous cycle stage had a greater influence on translatomic response to repeated ketamine in an MSN subtype-specific manner, occurring almost exclusively in D1- but not D2-MSNs in proestrus females and primarily related to regulation of transcription and posttranscriptional mRNA modification. These effects were observed several days after the final ketamine dose upon re-exposure to the drug-paired context, suggesting enduring cycle-specific ketamine-induced neuroadaptations in D1-MSNs.

The greater sensitivity of female rats to the locomotor-activating effects of repeated ketamine is consistent with earlier reports (Strong et al., 2017; Schoepfer et al., 2019) and parallels their increased behavioral responsiveness to ketamine's reinforcing properties (Strong et al., 2019; Wright et al., 2019). Among females, proestrus rats developed a greater enhancement of locomotor response to ketamine with fewer treatments than diestrus females. This cycle bias in behavioral responsiveness to ketamine is supported by the maintenance of low-dose ketamine self-administration in proestrus but not diestrus rats (Wright et al., 2017), together with the present work suggesting that in addition to sex, estrous cycle at the time of drug administration may confer differential sensitivity to ketamine's abuse-like potential. Interestingly, induction of locomotor sensitization to ketamine was not observed in males regardless of dose, contrasting with previous reports reporting sensitization development in this sex (Rocha et al., 2017; Trujillo and Heller, 2020; Elersič et al., 2024) at doses comparable to the highest tested here (20 mg/kg). While the reasons for this discrepancy are unclear, several factors influence locomotor sensitization to ketamine in males—including dose, treatment frequency, enantiomeric form, context, stress, and strain (Trujillo and Heller, 2020; Elersič et al., 2025). Furthermore, while no progressive increase in activity with repeated treatments was observed in males under this treatment regimen, expression of sensitized locomotor response to a lower ketamine dose was not determined after a prolonged drug-free period in the present work. It is therefore not possible to discount the possibility of a sensitized behavioral response in males if expression were to have been assessed at a later timepoint [Strong et al., 2017; Schoepfer et al., 2019; however, also see Simmler et al. (2022)].

For locomotor sensitization, the enhanced activity that occurs with repeated drug treatments relies upon both glutamatergic (Pulvirenti et al., 1991) and dopaminergic (Kelly and Iversen, 1976) signaling in the NAc. Within the NAc, activity and transcriptional response of D1-MSNs, in particular, modulate acute and sensitized locomotor response to psychostimulants (Savell et al., 2020; Van Zessen et al., 2021), at times in a sex-specific manner (Teague et al., 2024). Accordingly, we show here that repeated ketamine treatment uniquely alters D1-MSNs in female rats in an estrous cycle-dependent manner. Paralleling the faster and more robust sensitized response during proestrus versus diestrus, 46 DEGs were identified in D1-MSNs in proestrus, compared with only 9 in the same cell type in diestrus. These changes occurred on a background of estrous cycle-dependent differences between D1- and D2-MSN translatomes at baseline, providing some basis for differential treatment response within these cell populations. It should be briefly noted that as the focus of the present work aimed to determine molecular adaptations associated with the early induction of behavioral plasticity, sequencing of NAc D1- and D2-MSNs was only performed in female rats (who displayed such development) and not in males. While beyond the scope of the present work, examination of the neural correlates of potential sensitization expression in both males and females may be warranted to compare treatment response in MSNs of both sexes. Furthermore, while previous work in mice found that cell-type-dependent differences in the NAc of each sex were much more robust than sex differences within D1- and D2-MSN-enriched genes (Kronman et al., 2019), modest differences in MSN subtype translatomes at baseline may still be relevant to molecular adaptations of behavioral importance emerging with repeated ketamine treatment and are worthy of future investigation.

In D1-MSNs, most genes downregulated by ketamine represent either IEGs or LRGs induced by neuronal activation to guide future neuroplasticity (Phillips et al., 2023a). Interestingly, many of the IEGs downregulated by repeated ketamine (e.g., *Fosb*, *Fosl2*, *Peli1*, *Tac1*, *Dusp14*, *Nr4a3*) are induced in D1-MSNs by other addictive substances such as cocaine (Savell et al., 2020; Phillips et al., 2023b), suggesting alternative mechanisms at D1-MSNs underlying increased behavioral responsiveness to ketamine. Indeed, surprising negative regulatory roles of NAc D1-MSNs in cocaine reward and reinforcement have been identified (Zhao et al., 2022), perhaps owing to functionally distinct subpopulations of striatal D1-/D2-MSNs (Phillips et al., 2023b). Furthermore, as tissue in the present work was collected 4–5 d after the last ketamine dose, the observed IEG/LRG downregulation in ketamine-treated females may reflect compensatory reductions during a period of abstinence (Yan et al., 2023) or transcriptional rebound following drug-mediated IEG induction, as previously described following intravenous ketamine self-administration in rats (Kim et al., 2022). Nonetheless, future investigations implementing a chemogenetic approach to manipulate NAc D1-MSN activity alongside intermittent ketamine treatment during both estrous cycle stages may help to resolve the behavioral relevance of these enduring alterations in D1-MSNs selectively observed in proestrus females.

Alternative transcriptional and posttranscriptional mechanisms also emerged, with several transcription factors exhibiting increased translation in ketamine-treated proestrus D1-MSNs, including *Smad3*—an activin/transforming growth factor-β signaling effector (Massagué et al., 2005) implicated in both antidepressant-like response (Gergues et al., 2021) and relapse to cocaine (Gancarz et al., 2015). Smad3 also regulates generation of the mRNA splice variant encoding ΔFosB in cooperation with the RNA-binding protein, PCBP1, via a D1R-dependent alternative splicing mechanism in NAc MSNs that is functionally linked to sensitized locomotor response (Krapacher et al., 2022). Notably, translation of *Pcpb1* was greater in D1- versus D2-MSNs at baseline selectively during proestrus in our data, demonstrating one putative mechanism by which cell-type-specific regulation of MSN translatomes by the estrous cycle may influence susceptibility to drug-induced posttranscriptional events. While lower levels of translating *Fosb* were identified in D1-MSNs several days after ketamine treatment, potential drug-induced increases cannot be discounted. Furthermore, greater gene expression and translation are not necessarily observed alongside elevated ΔFosB levels following periods of withdrawal, likely owing to the stability of the ΔFosB protein (Alibhai et al., 2007). Alternatively, Smad3/PCBP1 may cooperatively regulate alternative splicing of additional targets and warrants future investigation.

Several DEGs upregulated in proestrus D1-MSNs by ketamine, including Smad3, also participate in m6A mRNA methylation (Bertero et al., 2018), a dynamic posttranscriptional mechanism responsive to NMDAR stimulation (Gowda et al., 2022) that regulates mRNA stability and degradation and is implicated in substance use and other neuropsychiatric disorders (Liu and Zhang, 2022; Kanarik et al., 2025). While its role in m6A methylation within the brain is unclear, Smad3 facilitates recruitment of the m6A methyltransferase complex (MTC) onto nascent RNAs and interacts with the MTC component Zc3h13 (Bertero et al., 2018), which was also upregulated by ketamine and facilitates nuclear localization of the MTC (Wen et al., 2018). These changes observed in proestrus D1-MSNs paralleled increases in *Prrc2c* and *Rbm3*—m6A-associated RNA-binding proteins with established roles in RNA stability (Wang et al., 2014; Xia et al., 2018), local protein synthesis (Smart et al., 2007), and synaptic plasticity (Sertel et al., 2021). Together, these findings support a novel D1-MSN-specific posttranscriptional program by which ketamine may regulate adaptive plasticity in an estrous cycle-dependent manner. As an avenue of future exploration, cell-type-specific manipulation of the above-described transcriptional regulators and/or m6A-related genes associated with repeated ketamine in NAc D1-MSNs would provide further insight into the functional importance of these changes to the early development of behavioral plasticity with intermittent treatment in rats of both sexes.

While all females developed sensitization to ketamine, it is unclear why only proestrus females exhibited persistent neuroadaptive changes in D1-MSNs—though these changes, too, were modest in number. It is possible that sensitization-associated changes are not solely reflected at the level of the NAc but rather are driven upstream through connected regions, including the medial prefrontal cortex (mPFC), known to affect locomotor sensitization to psychostimulants (Tzschentke and Schmidt, 1998). However, previous work conducted in male and female mice failed to demonstrate drug-evoked plasticity at mPFC-to-NAc D1-MSN synapses following repeated ketamine treatment (Simmler et al., 2022). The NAc also receives glutamatergic input from regions such as the hippocampus (Lodge and Grace, 2008) and thalamus (Clark et al., 2017) with evidenced roles in locomotor sensitization (Lodge and Grace, 2008) that may be of interest as alternative targets. While the NAc does receive modulatory dopaminergic input from the ventral tegmental area, the effects of both acute (Simmler et al., 2022) and repeated (Iro et al., 2021; Simmler et al., 2022) ketamine on synaptic plasticity within this area demonstrate only transient, but not sustained, effects of treatment. However—noting that the changes identified in proestrus were observed several days after the final treatment—the minimal effect of ketamine on diestrus MSNs at this timepoint might also reflect a more transient sensitized locomotor response compared with proestrus females, given that the persistent neuroadaptations emerging after treatment cessation are those that influence enduring changes in future responsiveness to the drug (or drug context; Kalivas, 2003). Unfortunately, as expression of sensitization was not determined, it cannot be concluded that these neuroadaptations predict a long-lasting maintenance of sensitized response during proestrus. Nonetheless, together with our previous work (Wright et al., 2017), these findings suggest that sex and potentially hormonal status may influence the addictive potential of ketamine—in part, through enduring molecular adaptations in NAc D1-MSNs.

On the other hand, these findings in diestrus females and the lack of reinforcing properties during this cycle stage (Wright et al., 2017) suggest that the addictive potential of ketamine may not be sufficiently high in some individuals to cause concern for long-term liability under protracted low-dose administration. Furthermore, though ketamine is reinforcing in males when administered contingently once every 4 d (Wright et al., 2017), noncontingent administration at this interval in the present work—which more closely resembles clinician-administered treatment protocols for depression—failed to confer the same liability when treatment was separated from motivation to take the drug. In line with this, increased effort requirements attenuate ketamine's reinforcing efficacy in mice, an effect parallelled by a transient increase in dopaminergic tone in the NAc without the adaptive plasticity typically evoked by repeated psychostimulant administration (Simmler et al., 2022). Thus, the rewarding and reinforcing properties of ketamine short-term do not necessarily confer future dispositional liability toward abuse or induce drug-adaptive plasticity. Future investigations should aim to resolve why sensitized proestrus females uniquely display enduring alterations in D1-MSNs following repeated low-dose ketamine treatment.

Overall, we found that the greater susceptibility of female rats to develop sensitization to intermittent ketamine was associated with enduring estrous cycle-dependent neuroadaptations primarily in NAc D1-MSNs. Furthermore, we have identified cell-type-specific posttranscriptional mechanisms in the NAc through which repeated ketamine may produce enduring changes in behavior. However, given the lack of effect of ketamine treatment on NAc MSNs in diestrus females despite apparent sensitization, it is unclear whether these mechanisms portend future addictive liability or whether they may be relevant to other reward or affect-related behaviors. These findings encourage further examination into the effects of ketamine on brain plasticity and the safety of using chronic ketamine for the treatment of various psychopathologies.

## Synthesis

Reviewing Editor: Christie Fowler, University of California Irvine Decisions are customarily a result of the Reviewing Editor and the peer reviewers coming together and discussing their recommendations until a consensus is reached. When revisions are invited, a fact-based synthesis statement explaining their decision and outlining what is needed to prepare a revision will be listed below. The following reviewer(s) agreed to reveal their identity: NONE. Note: If this manuscript was transferred from JNeurosci and a decision was made to accept the manuscript without peer review, a synthesis may not be available.

This manuscript was transferred from JNeuro following reviewer comments and author responses, with revision modifications denoted. The studies examined the effects of ketamine on locomotor sensitization and related transcriptional changes in D1 and D2 neurons in the nucleus accumbens. Interestingly, estrous cycle was found to influence the development of ketamine sensitization, which was also reflected in transcriptional changes in the nucleus accumbens medium spiny neurons. The response to the reviewers was found to be appropriate and well justified. While the findings are largely correlational and could be attributed to either ketamine's actions and/or the differences in locomotion, the sequencing data set provides valuable information to support future studies in the field.

For publication, it is advisable to be consistent with the supplementary files and not submit photoshop files. Thus, Fig S1 should be a pdf, whereas the Tables should all be xls.

Congratulations on your publication!
