# Supplementary figures and images for: Estrous Cycle Influences Cell-Type-Specific Translatomic Signatures of Repeated Ketamine Exposure in the Rat Nucleus Accumbens
Source: eNeuro. 2026 Jan 15;13(1):ENEURO.0419-25.2025. doi: 10.1523/ENEURO.0419-25.2025 (PMC12826133; doi:10.1523/ENEURO.0419-25.2025)

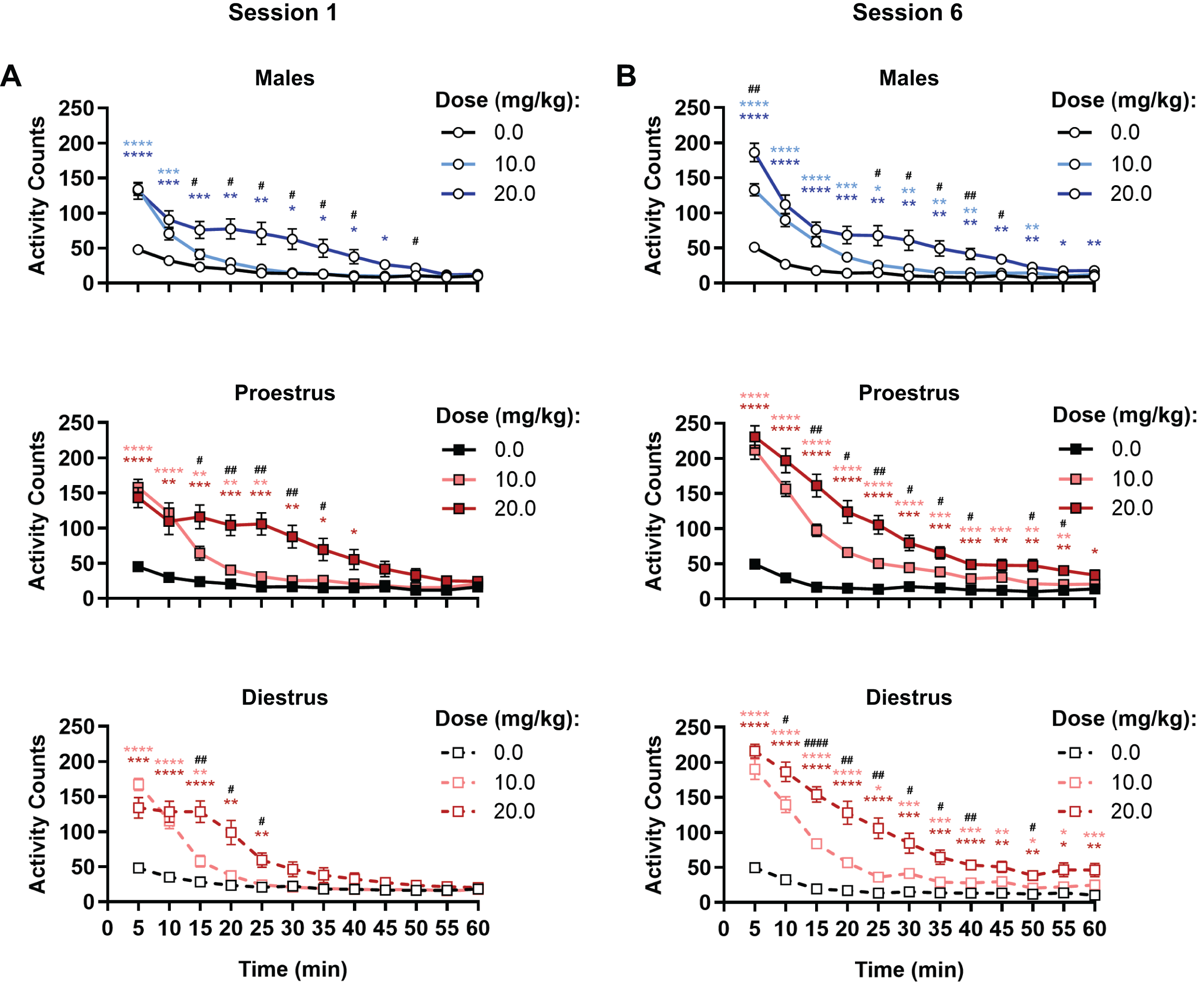

Supplement: Figure 3-1 — Dose comparison of temporal locomotor response patterns for first and last ketamine treatments within sex and estrous cycle stage. A, Males (top), proestrus females (middle), and diestrus females (bottom) exhibit dose-dependent increases in activity most notably during the first 25 min after a single 10.0 (light blue, pink asterisks) or 20.0 (dark blue, red asterisks) mg/kg ketamine injection compared to vehicle. B, Between-dose comparisons after the sixth ketamine injection in males (top), proestrus females (middle) and diestrus females (bottom) demonstrate extended increases in activity compared to saline across the hour-long session. Data are expressed as means ± SEMs; *p < 0.05, **p < 0.01, ***p < 0.001, ****p < 0.0001 vs. 0.0 mg/kg; #p < 0.05, ##p < 0.01, ###p < 0.001, ####p < 0.0001, 20.0 mg/kg vs. 10.0 mg/kg; Tukey’s multiple comparisons. Download Figure 3-1, TIF file. [file eneuro-13-ENEURO.0419-25.2025-s002.tif]
